# Assessing Planning Ability Across the Adult Life Span in a Large Population-Representative Sample: Reliability Estimates and Normative Data for the Tower of London (TOL-F) Task

**DOI:** 10.1017/S1355617718001248

**Published:** 2019-05

**Authors:** Josef M. Unterrainer, Benjamin Rahm, Christoph P. Kaller, Philipp S. Wild, Thomas Münzel, Maria Blettner, Karl Lackner, Norbert Pfeiffer, Manfred E. Beutel

**Affiliations:** 1 Medical Psychology and Medical Sociology, Faculty of Medicine, University of Freiburg, Freiburg, Germany; 2 Department of Neuroradiology, University Medical Center Freiburg, Freiburg, Germany; 3 Preventive Cardiology and Preventive Medicine, Center for Cardiology, University Medical Center of the Johannes Gutenberg-University Mainz, Mainz, Germany; 4 Center for Thrombosis and Hemostasis, University Medical Center of the Johannes Gutenberg-University Mainz, Mainz, Germany; 5 Center for Translational Vascular Biology (CTVB), University Medical Center of the Johannes Gutenberg-University Mainz, Mainz, Germany; 6 German Center for Cardiovascular Research (DZHK), partner site RhineMain, Mainz, Germany; 7 Center for Cardiology – Cardiology I, University Medical Center of the Johannes Gutenberg-University Mainz, Mainz, Germany; 8 Institute of Medical Biostatistics, Epidemiology and Informatics, University Medical Center of the Johannes Gutenberg University Mainz, Mainz, Germany; 9 Institute of Clinical Chemistry and Laboratory Medicine, University Medical Center of the Johannes Gutenberg University Mainz, Mainz, Germany; 10 Department of Ophthalmology, University Medical Center Mainz, Mainz, Germany; 11 Department of Psychosomatic Medicine and Psychotherapy, University Medical Center of the Johannes Gutenberg University Mainz, Mainz, Germany

**Keywords:** Tower of London, Planning, Reliability, Normative data, TOL-F, Gutenberg Health Study (GHS)

## Abstract

**Objectives:** The Tower of London (TOL) test has probably become the most often used task to assess planning ability in clinical and experimental settings. Since its implementation, efforts were made to provide a task version with adequate psychometric properties, but extensive normative data are not publicly available until now. The computerized TOL-Freiburg Version (TOL-F) was developed based on theory-grounded task analyses, and its psychometric adequacy has been repeatedly demonstrated in several studies but often with small and selective samples. **Method:** In the present study, we now report reliability estimates and normative data for the TOL-F stratified for age, sex, and education from a large population-representative sample collected in the Gutenberg Health Study in Mainz, Germany (*n*=7703; 40–80 years). **Results:** The present data confirm previously reported adequate indices of reliability (>.70) of the TOL-F. We also provide normative data for the TOL-F stratified for age (5-year intervals), sex, and education (low *vs*. high education). **Conclusions:** Together, its adequate reliability and the representative age-, sex-, and education-fair normative data render the computerized TOL-F a suitable diagnostic instrument to assess planning ability. (*JINS*, 2019, *25*, 520–529)

## INTRODUCTION

Since the introduction of the Tower of London (TOL) planning paradigm by Tim Shallice (1982), several studies have denoted insufficient psychometric properties of the task, especially an insufficient reliability of the original 12-item problem set (*α*=.25; Humes, Welsh, Retzlaff, & Cookson, 1997; see also Berg & Byrd, 2002; Kafer & Hunter, 1997; Lowe & Rabbitt, 1998).

One approach to increase its reliability was to select items from a larger pool of items based on the item-total correlations and to re-evaluate the internal consistency of the resulting set of items (cf. Schnirman, Welsh, & Retzlaff, 1998; 30-item TOL with *α*=.79). As an alternative approach, based on comprehensive problem space analyses and empirical data, Kaller, Unterrainer, and Stahl (2012) introduced a TOL problem set of 32 items consisting of four-, five-, six-, and seven-move TOL problems. This version revealed acceptable split-half reliability (*r*=.72) and internal consistency (*α*=.69) values for TOL performance in terms of the total number of correctly solved problems.

Further improvement through item selection resulted in the development of the computerized TOL-Freiburg Version (TOL-F; Kaller, Unterrainer, Kaiser, Weisbrod, & Aschenbrenner, 2012), with a substantial reduction from 32 to 24 items using four- to six-move TOL problems only. Subsequently, Kaller et al. (2016) presented psychometric data on the TOL-F from two large-scale samples revealing adequate internal consistency and split-half reliability (*α*=.73; ω_tot_=.73; glb=.76) both of which were stable across the adult life span. In summary, TOL versions are now available that provide satisfactory reliability, a sufficiently broad range of item difficulties and an adequate test economy in terms of a relatively short and clinically practicable test duration.

From this overview, it seems as if test versions that comprised a larger number of problems yielded higher reliability. One reason for this may be that the abovementioned studies by Schnirman et al. (1998) and our own group that provided adequate test criteria have used an optimized selection of problems drawn from a larger item pool. But moreover, a larger number of problems may be advantageous in that it reduces the impact of basic strategy learning during early parts of testing on overall performance. For example, results by Shallice (1982) and especially Morris, Miotto, Feigenbaum, Bullock, and Polkey (1997), the latter using the Tower-of-Hanoi, suggested that early items may stress different processes when the participant is developing a strategy from later problems where strategy may be relatively stable. Quite obviously, stable strategy should result in more stable estimates of planning performance.

While it has become common to publish a detailed description of the single items used in a study (e.g., Culbertson & Zillmer, 1998; Krikorian, Bartok, & Gay, 1994), supporting tests of reproducibility and the comparability of different versions, there is a clear shortage of publicly available normative data. As a notable exception, Rognoni et al. (2013) presented normative data of Spanish young adults (age 18 to 49 years; *n*=179) of the 10-item Tower of London-Drexel University test (Culbertson & Zillmer, 2001). Michalec et al. (2017) provided normative standards of 298 healthy adults (age 19 to 84 years) using the original 12-item TOL.

Only recently, Boccia et al. (2017) reported the standardization of a 16-item TOL (containing the original 12 items by Shallice plus 4 newly added problems; *n*=896 individuals, aged 15–86 years), taking into account gender, age, and years of education. This was well justified by previous findings showing that planning ability clearly depends on age, education level, and sex (D’Antuono et al., 2017). Kaller et al. (2016) revealed a linear increase of difficulty, sex, and age. To be specific, performance differences between the sexes and the age groups gradually increased from four-, over five-, to six-move problems. This is in line with larger performance variability in more difficult problems, whereas easier four-move problems are usually almost perfectly solved by most participants.

Yet, in accordance with the Board of Assessments of the European Federation of Psychologists’ Associations (EFPA, 2013), good to excellent sample sizes in subgroups should contain 100 to 150 respondents each. Although the overall number of approximately 900 individuals in the study of Boccia et al. is quite respectable, it is clearly insufficient given this recommendation for fine-grained analyses. In some subgroups, percentiles were not applicable due to the limited number of participants (Boccia et al., 2017).

As outlined above, a psychometrically well-validated and reliable TOL version providing a fine-grained standardization with a large sample size has not been available by now, but would be highly desirable for use of the TOL by neuropsychologists in both research and clinical practice. Thus, the aim of the present study was two-fold: To re-evaluate the reliability of the previously reported TOL version across the adult life span in a larger sample and to use a sufficiently large number of participants to provide normative data that account for individual age, education level, and sex. We believe that there is a common agreement in test development to adjust for sociodemographic data such as age or educational attainment (see, e.g., Wechsler Adult Intelligence Scale-Revised, or Raven tests). This renders comparisons within groups more meaningful, which may be especially important for clinical assessments, and often is regarded as increasing test fairness for comparisons across groups.

As the only publicly available standardization of the TOL by Boccia et al. (2017) also adjusted for age, education level, and sex, we followed their approach, however, providing the recommended number of cases and a psychometrically improved TOL version. To these aims, we present psychometric and normative data on the computerized TOL-F (24 items) from a large sample (*n*=7703) collected in the Gutenberg Health Study (GHS) in Germany.

## METHODS

### Sample

The GHS was designed as a population-based, prospective, observational, single-center cohort study in the Rhine-Main region in western mid-Germany. The primary aim was to evaluate and improve cardiovascular risk stratification. The still-ongoing project examines cardiovascular diseases, cancer, eye diseases, metabolic diseases, diseases of the immune system, and mental diseases. The study aims at improving the individual risk prediction for diseases. Therefore, lifestyle, psychosocial and environmental factors, laboratory parameters, as well as the extent of the subclinical disease are investigated.

In the baseline examination between April 2007 and March 2012, the GHS assessed a representative population sample of approximately 15,000 individuals from the city of Mainz and the district of Mainz-Bingen (overall population approximately 400,000 residents). The sample was drawn randomly from the governmental local registry offices in the city of Mainz and the district of Mainz-Bingen, where every inhabitant of the area is obliged to register. The sample was stratified 1:1 for sex and residence (urban and rural) and in equal strata for decades of age. Individuals between 35 and 74 years of age were enrolled, and written, informed consent was obtained from all participants. No seeding of persons with very low ability or health status was performed. The only exclusion criteria concerned insufficient knowledge of the German language to understand instructions and to give informed consent and physical or psychological inability to participate in the examinations at the study center. The norms thus are based on data of German speakers of different backgrounds. Demographic characteristics of the sample are presented in Table 1.

**Table 1.** Demographic characteristics of the Gutenberg Health Study sample

	All (7703)	Men (51.4%)	Women (48.6%)
Age [years]	59.5 (10.6)	59.8 (10.6)	59.2 (10.5)
Body mass index [kg/m²]	26.9 (24.1/30.2)	27.4 (25.1/30.3)	26.0 (23.1/30.1)
SES*			
Not retired	14.06 (4.20)	14.59 (4.22)	13.42 (4.09)
Retired	11.41 (4.35)	12.16 (4.44)	10.70 (4.14)
Education			
Years of education	12.90 (2.01)	13.16 (2.07)	12.63 (1.91)
Secondary schools	35.9%	35.9%	36.0%
No vocational training	5.4%	2.6%	8.3%
Apprenticeship	46.9%	38.2%	56.0%
Technician/master	15.6%	19.0%	12.0%
Intermediate secondary schools	24.9%	18.8%	31.3%
High school	10.2%	13.6%	6.6%
University, university of applied science	11.3%	15.8%	6.5%
Marital status			
Living in a partnership	86.1%	89.0%	82.9%
Married	74.5%	78.3%	70.5%
Married, living separated	1.8%	1.8%	1.9%
Registered partnership	0.1%	0.1%	0.1%
Divorced	9.0%	7.8%	10.2%
Widowed	6.1%	2.9%	9.6%
Unmarried	8.4%	9.1%	7.8%
Status of employment			
Unemployed	2.2%	2.6%	1.8%
Full-time	40.5%	53.3%	27.0%
Part-time	12.2%	3.4%	21.6%
Small-scale employment	3.5%	2.2%	4.9%
Retired	42.4%	39.5%	45.5%
Income [€, after tax]			
Not retired	2125 (1375/3375)	2875 (1979/3875)	1625 (875/2125)
Retired	1375 (875/2125)	1875 (1375/2875)	875 (450/1625)
Household income [€, after tax] Not retired			
<1250	3.7%	2.7%	5.0%
1250–2500	19.9%	16.7%	23.7%
>2500	76.4%	80.6%	71.3%
Household income [€, after tax] Retired			
<1250	8.7%	6.2%	11.2%
1250 - 2500	40.1%	37.8%	42.4%
>2500	51.2%	56.0%	46.4%

* Socioeconomic status (SES) was defined according to Lampert and Kroll’s scores of SES (Lampert & Kroll, 2006) ranging from 3 to 21 with 3 indicating the lowest and 21 the highest SES. This scoring combines three different dimensions that represent school education level and professional training, income, and professional status. Please note that all variables that comprise the participants’ income are indicated separately for not retired and retired subjects. Normally distributed variables are presented by their mean and their standard deviation (one number in brackets). Variables not following a normal distribution are shown using their median and their interquartile range (two numbers in brackets). Relative frequencies are shown in percent.

The present analyses comprise 7870 subjects who participated in the second run of the GHS and were tested between June 2012 and December 2015. Subjects’ age ranged between 40 and 80 years.

The GHS was approved by local ethics authorities. Data acquisition complied with local institutional research standards for human research and was completed in accordance with the Helsinki Declaration.

To assess effects of age on planning ability, the sample was divided into eight 5-year groups between 40 and 80 years of age, covering an age range from mid- to late adulthood (Table 2). Besides individual age and sex, subjects were also characterized by their highest achieved education level assessed on a 5-point scale with the following levels (Kaller et al., 2016): An educational level of 1 corresponded to 8 or less years of schooling and was typically applied to participants who completed elementary school, but did not obtain higher education (*n*=73). An educational level of 2 was used to classify participants who completed 9 years of schooling, but without vocational training (*n*=798). An educational level of 3 corresponded to 10 to 12 years of education and the completion of vocational training (*n*=3956). An educational level of 4 was used to denote the completion of high school and the qualification for university entrance (*n*=809). An educational level of 5 was assigned if a participant had obtained an academic degree (*n*=2241). Information on education level was not available for two subjects who were consequently excluded.

**Table 2.** Descriptives of the Gutenberg Health Study sample

Sex	ED.L.	Age groups (years)	*N*	Accuracy (*mean*)	Accuracy (*SD*)	Cancel 3 False (%)	Time out 20 min (%)
Male	Low	40.00–44.99	121	15.62	3.5	4.1	1.7
		45.00–49.99	205	14.77	3.5	3.9	1.5
		50.00–54.99	274	14.80	3.2	5.1	1.1
		55.00–59.99	273	13.95	3.2	7.7	1.5
		60.00–64.99	313	13.74	3.4	13.1	2.9
		65.00–69.99	312	12.56	3.4	16.7	3.8
		70.00–74.99	357	11.61	3.4	24.9	7.3
		75.00–79.99	247	11.04	3.8	24.3	11.3
	High	40.00–44.99	226	16.33	3.2	4.9	0.4
		45.00–49.99	249	16.36	3.3	4.0	0.4
		50.00–54.99	316	15.56	3.3	5.4	0.3
		55.00–59.99	287	15.27	3.3	9.1	0.7
		60.00–64.99	251	14.62	3.2	8.0	0.8
		65.00–69.99	216	13.67	3.6	19.4	2.3
		70.00–74.99	175	12.30	3.1	20.0	7.4
		75.00–79.99	140	11.35	3.7	28.6	14.3
Female	Low	40.00–44.99	171	14.40	3.2	4.7	0.0
		45.00–49.99	284	14.13	3.1	6.7	0.7
		50.00–54.99	291	13.57	3.4	9.6	1.7
		55.00–59.99	361	13.48	3.3	16.1	1.4
		60.00–64.99	398	12.23	3.3	16.8	2.8
		65.00–69.99	395	11.77	3.3	23.3	4.8
		70.00–74.99	413	10.54	3.4	36.3	6.5
		75.00–79.99	254	9.69	3.2	39.0	14.6
	High	40.00–44.99	176	15.56	3.0	5.1	0.0
		45.00–49.99	235	15.23	3.0	6.0	0.9
		50.00–54.99	208	14.51	3.7	11.1	1.4
		55.00–59.99	195	13.62	3.1	9.7	1.0
		60.00–64.99	123	13.02	3.4	18.7	1.6
		65.00–69.99	104	12.51	3.3	26.9	6.7
		70.00–74.99	83	12.14	3.4	27.7	6.0
		75.00–79.99	50	11.00	3.6	32.0	12.0

*Note*. Sample descriptives in dependence of sex, education level (ED.L.), and age. N denotes the respective subsample size. Accuracy represents the total number of correctly solved TOL-problems (at maximum 24). Test cancellations due to exceeding the item-wise time limit for solution three times in a row (Cancel 3 False) are given as percentage of the subsample. Likewise, test cancellations because of reaching the overall 20-min limit for the duration of the test session (Time out 20 min) are presented as percentage of the subsample.

To preserve sufficient numbers of subjects within cells considering the factors sex and age groups, we excluded participants with educational level 1 (0.93% of the overall sample) from further analysis and merged participants with educational level 2 and 3 to a factor labeled “low education” and participants with educational level 4 and 5 to one factor called “high education”.

Data inspection revealed 73 cases (0.99%) of the sample with no usable data that presumably showed a lack of motivation or task compliance and that were hence also excluded before the analyses. At the beginning of the study, participants used a computer mouse to solve the tasks. Due to handling-problems in older subjects, the study continued with a touchscreen as response device. Thus, the first 19 cases who had used the computer mouse were excluded and the final sample consisted of *n*=7703 participants.

An overview on the descriptive information for age, sex, and education level of the two overall samples as well as of the resulting subgroups is provided in Table 2.

### Tower of London – Freiburg Version (TOL-F)

#### Task description

The TOL-F (Kaller, Unterrainer, Kaiser, et al., 2012) is as a computerized pseudo-realistic representation of the originally wooden configuration of the Tower of London and is implemented in the Vienna Test System (VTS; https://www.schuhfried.com/test/TOL-F, last accessed 2018-04-18).

In the TOL-F, individual problem items consist of a start and a goal state that are presented in the lower and upper halves of the computer screen, respectively. Subjects are instructed to transform the start into the goal state in the minimum number of moves which are shown to the left of the start state. Written instructions inform that only one ball may be moved at a time, that balls cannot be placed beside the rods, that only the top-most ball can be moved in case several balls are stacked on a rod, and that the rods differ in their capacities of accommodating one, two, or three balls at maximum. The computer program does not allow breaking these rules, but records any attempts to do so. Instructions further emphasize that problems have to be solved in the minimum number of moves and that participants should always plan ahead the problem solution before starting with movement execution.

To transfer the start into the goal state, the TOL-F can be worked on by touch screen. Thus, a ball is picked up simply by clicking the ball *via* finger touch. The selected ball is then encircled by a transparent whitish corona and can be moved to another rod. The respective rod is likewise selected by finger touch. Participants were not allowed to retract moves after they were made.

During the instruction phase, participants’ task comprehension was controlled by two two-move problems. To get used to the task and to handling the touchscreen, participants practiced with an additional set of four three-move problems. Only thereafter, the proper testing started, comprising eight four-, five-, and six-move problems presented in increasing minimum number of moves, respectively. The instruction and practice phase was scheduled to take 5 min, whereas for the testing of the 24 problems a time limit of 20 min was applied. After initial pilot testing in 2012, it turned out that this time limit was sufficient for most participants. In a previously published report on a subsample of the present one (*n*=3770; Kaller et al., 2016) 95% of the participants finalized the overall task (inclusive instructions) after 22 min. Thus, in most cases the pre-specified time was sufficient.

In addition, a 1-min time limit per trial was implemented, like in the original study of Shallice (1982). To avoid unnecessary frustration (and a reduced compliance and/or motivation in subsequent tests, for instance, in a clinical setting), the TOL-F allows for an automatic cancellation of the test if the time limit of a single trial is exceeded three times in a row. In Table 2, right column, the percentage of test cancellations due to exceeding time limits after three times is presented as a function of age, education level, and sex. As becomes obvious, cancellation rate considerably increased from 40 to 80 years.

As for the automatic cancellation of the test if the time limit of a single trial was exceeded three times in a row, the percentage of participants who failed to finalize the test session within 20 min clearly increased with age (Table 2, rightmost column). Statistical analyses did not reveal any biases depending on educational level, that is, time out rate was not increased in older participants with low compared to high education. Further details of the experimental procedure and the problem set used are described in Kaller et al. (2016).

The TOL-F was the only cognitive test, and thus the only digitally provided test, during the GHS-procedure. It was embedded in a series of non-cognitive medical examinations comprised in the GHS.

#### Dependent measures

For assessment of individual planning ability with the TOL-F, overall planning accuracy, defined as the percentage of problems that were correctly solved in the minimum number of moves, is regarded as the primary outcome variable of interest. The TOL-F provides three different levels of minimum moves (four-, five-, and six-move problems, eight of each) resulting in an overall planning accuracy of max. 24 problems.

### Data Analyses

#### Analyses of variance

Analyses of variance (ANOVAs) on planning accuracy as dependent variable were conducted using IBM SPSS Statistics for Windows (Version 23.0.0.2) to test for main effects and interactions of the between-subjects factors *Age Group, Education Level,* and *Sex*.

#### Reliability estimates

In accordance with the study of Kaller et al. (2016) and based on the revised review model for the description and evaluation of psychological and educational tests (Version 4.2.6; http://www.efpa.eu/professional-development/assessment) recently suggested by the Board of Assessments of the European Federation of Psychologists’ Associations (EFPA, 2013), the following estimates of reliability are reported: Lambda 2 (λ2), lambda 3 (λ3) reflecting Cronbach alpha (α), lambda 4 (λ4), omega total (ω_tot_), and the greatest lower bound (glb).

While all these indices seek to provide estimates of the lower bound of true test reliability, they differ with respect to their exact assumptions and their computation. Guttman’s lambda 3 reflects the mean of all split-half reliabilities, but is said to often underestimate true reliability (Revelle & Zinbarg, 2009; Sijtsma, 2009). Compared to lambda 3, lambda 2 additionally takes into account inter-item covariance. As the sum of squares of covariances is used, lambda 2 will in the vast majority of cases be higher than lambda 3 but never lower (Guttman, 1945). Lambda 4 is calculated by dividing the total pool of items into two halves in such a way that the covariance between scores on the two halves is as high as possible, it should thus represent the greatest split-half reliability that can be attained.

Sijtsma (2009) recommended the glb as the best estimate of the lower limit of true reliability. Based on classical test theory, observed scores are considered as the sum of the true covariance matrix between items and the diagonal matrix of item error covariances. Estimating the glb is then pursued by finding the error matrix whose sum of diagonal elements is maximum, while both the resulting true item covariance matrix and the error covariance matrix are still valid (that is, non-negative definite) covariance matrices (Bendermacher, 2017).

Revelle and Zinbarg (2009) favored the alternative estimate omega that represents the total reliable variance estimated by a factor model as it may often be closer to the true value than glb, and often reaches higher values. In their study, glb actually never provided the highest estimate.

Only recently, Tunstall, O’Gorman, and Shum (2016) published reliability estimates on a Tower of London version. In addition to Cronbach’s alpha, they also provided lambda 4 (λ4), omega total (ω_tot_), and the glb. Reporting these indices here thus additionally facilitates comparisons to the present findings.

All indices were computed for the overall sample as well as for the respective age subgroups using the *psych* package (Version 1.3.2; Revelle, 2013) for the *R* open-source statistical software (Version 3.4.3; R Core Team, 2013).

#### Normative data

Normative data in the tables contain rounded raw cumulative percentages sorted by the total number of correctly solved problems. No z-transformation or smoothing was applied.

## RESULTS

### Effects of Age, Education Level, and Sex on Planning Accuracy

An ANOVA with the between-subjects factors *Age Group* (eight 5-year intervals), *Education Level* (low *vs*. high), and *Sex* (male *vs*. female), and planning accuracy as dependent variable revealed significant main effects of *Age Group* (*F*_(7,7671)_=166.51; *p*<.001; *η* ^2^_partial_=.132), *Education Level* (*F*_(7,7671)_=124.43; *p*<.001; *η* ^2^_partial_=.016), and *Sex* (*F*_(1,7671)_=141.11; *p*<.001; *η* ^2^_partial_=.018). As evident from Table 2, planning accuracy decreased with age and was reduced in less educated as well as in female participants.

Beside these main effects, present data also reveal a significant three-way interactions of *Age Group* by *Education Level* and by *Sex* (*F*_(7,7671)_=2.43; *p*=.018; *η* ^2^_partial_=.002). Graphical analyses suggest that the mean difference of approximately one more solved problem in higher compared to lower educated participants is rather stable for men and women across the life span, however, with one exception: Women in the age group of 55 to 59.99 years revealed equal planning performances for both education levels, which should explain the significant triple-interaction. No other interactions reached significance (highest *F*=1.34; lowest *p*=.225).

### Reliability Estimates for Overall Planning Accuracy

Reliability estimates are provided in Table 3. The five different estimates of the overall sample on reliability ranged between .715 and .757. As in the preceding analyses of Kaller et al. (2016), in both the overall samples and in the respective age groups, estimates were highest for *glb* and *λ*_*4*_, whereas *λ*_*3*_ or Cronbach’s *α* yielded the lowest estimate in all cases.

**Table 3.** Reliability estimates of the Tower of London (TOL-F) task

(Sub)Sample	N	Age *M*±*SD* (yr)	Sex m, f (*N*)	ED. L. low, high (*N*)	λ_2_	λ_3_ (α)	λ_4_	ω_tot_	glb
Overall sample	7703	60.00±10.59	3962, 3741	4669, 3034	0.719	0.715	0.755	0.732	0.757
40.00–44.99 years	694	43.05±1.25	347, 347	292, 402	0.639	0.631	0.708	0.651	0.722
45.00–49.99 years	973	47.49±1.48	454, 519	489, 484	0.633	0.625	0.697	0.645	0.690
50.00–54.99 years	1089	52.43±1.46	590, 499	565, 524	0.668	0.656	0.717	0.674	0.726
55.00–59.99 years	1116	57.47±1.45	560, 556	634, 482	0.633	0.625	0.697	0.648	0.728
60.00–64.99 years	1085	62.50±1.42	564, 521	711, 374	0.669	0.661	0.735	0.681	0.730
65.00–69.99 years	1027	67.33±1.42	528, 499	707, 320	0.681	0.674	0.738	0.696	0.757
70.00–74.99 years	1028	72.52±1.4	532, 496	770, 258	0.687	0.679	0.744	0.704	0.752
75.00–79.99 years	691	77.18±1.42	387, 304	501, 190	0.737	0.729	0.797	0.754	0.802

*Note.* Reliability estimates for the overall sample and age-related subgroups on the TOL-F. *M*=mean; *SD*=standard deviation; yr=years; m=male; f=female; *N*=number of participants in the (sub-)sample.

### Standardization

Normative data for age groups and education level are provided in Table 4. Sex-adjusted versions of this table are presented in Tables 5 and 6 for women and men, respectively.

**Table 4.** Normative data of the Tower of London (TOL-F) task adjusted for age and education

	Low education								High education							
TOL	40–44 years	45–49 years	50–54 years	55–59 years	60–64 years	65–69 years	70–74 years	75–79 years	40–44 years	45–49 years	50–54 years	55–59 years	60–64 years	65–69 years	70–74 years	75–79 years
1	0	0	0	0	0	0	0	0	0	0	0	0	0	0	0	1
2	0	0	0	0	0	0	1	2	0	0	0	0	0	0	0	1
3	0	0	0	0	0	1	2	3	0	0	0	0	0	0	0	1
4	0	0	1	0	1	1	3	6	0	0	0	0	1	1	1	4
5	0	0	1	0	1	3	6	8	0	0	0	0	1	3	1	5
6	1	1	2	1	4	6	10	15	0	0	1	0	2	3	2	8
7	1	3	3	3	6	9	16	23	0	0	2	1	4	6	8	16
8	3	4	5	6	11	14	22	31	1	2	4	4	6	10	13	24
9	7	8	8	12	15	21	30	40	3	3	7	8	9	16	21	32
10	11	11	13	17	24	30	42	49	4	6	10	13	13	21	29	41
11	17	19	21	26	33	42	55	61	9	10	15	18	20	28	38	53
12	24	29	30	36	46	52	67	72	13	17	22	26	30	38	50	65
13	33	37	41	45	56	65	76	80	22	23	29	38	40	53	66	73
14	45	49	53	57	68	75	85	88	31	31	41	47	54	60	76	78
15	55	61	64	70	77	86	90	92	42	42	53	57	65	71	84	86
16	66	72	73	80	85	91	94	96	54	56	64	70	75	82	92	91
17	76	84	84	88	90	96	98	98	66	69	74	79	85	89	95	96
18	85	90	92	94	95	97	99	100	76	80	82	87	91	95	98	99
19	92	95	96	97	98	99	99	100	87	88	89	95	95	97	99	100
20	96	98	98	99	99	100	100	100	94	93	96	97	98	99	100	100
21	98	99	99	100	100	100	100	100	98	98	98	99	100	99	100	100
22	100	100	100	100	100	100	100	100	100	99	99	100	100	100	100	100
23	100	100	100	100	100	100	100	100	100	100	100	100	100	100	100	100
24	100	100	100	100	100	100	100	100	100	100	100	100	100	100	100	100
*N*	292	489	565	634	711	707	770	501	402	484	524	482	374	320	258	190

Percentile ranks of TOL performance, separately for low and high education and age groups. In the left-most column, the number of correctly solved TOL-problems is listed.

**Table 5.** Age and education adjusted normative data of the Tower of London (TOL-F) task for women

	Low education								High education							
TOL	40–44 years	45–49 years	50–54 years	55–59 years	60–64 years	65–69 years	70–74 years	75–79 years	40–44 years	45–49 years	50–54 years	55–59 years	60–64 years	65–69 years	70–74 years	75–79 years
1	0	0	0	0	0	0	0	0	0	0	0	0	0	0	1	0
2	0	0	1	0	0	0	1	1	0	0	0	0	0	0	1	2
3	0	0	1	0	0	1	2	3	0	0	0	0	0	0	1	2
4	0	0	1	0	1	2	4	6	0	0	0	0	1	2	2	6
5	0	0	1	1	2	4	6	10	0	0	1	0	1	3	4	6
6	1	1	2	1	6	7	12	18	0	0	2	0	2	3	4	10
7	1	2	4	4	9	10	19	27	1	0	5	3	7	7	11	12
8	2	4	6	7	13	16	26	36	1	2	7	6	11	11	13	26
9	6	8	11	13	19	24	36	47	3	4	11	10	15	16	18	36
10	12	11	18	19	29	33	48	55	5	7	14	17	21	25	29	42
11	19	20	26	29	39	47	62	70	10	13	18	24	31	38	39	56
12	29	31	34	39	55	58	74	81	15	21	29	34	41	49	52	70
13	41	40	48	48	65	71	81	88	26	28	36	51	55	63	64	74
14	53	52	62	59	78	78	90	95	38	37	46	62	66	71	76	80
15	63	64	71	72	83	88	93	97	47	49	60	71	76	84	87	88
16	71	76	79	82	89	93	96	99	59	63	70	79	86	90	93	94
17	81	88	89	89	94	96	98	100	72	77	77	90	92	94	95	96
18	89	94	95	94	97	98	99	100	83	88	84	95	94	98	98	100
19	95	98	98	97	99	99	100	100	92	94	93	97	98	98	99	100
20	98	99	98	99	99	100	100	100	95	97	98	98	99	98	100	100
21	99	99	99	100	100	100	100	100	98	99	99	100	100	99	100	100
22	100	100	100	100	100	100	100	100	100	99	100	100	100	100	100	100
23	100	100	100	100	100	100	100	100	100	100	100	100	100	100	100	100
24	100	100	100	100	100	100	100	100	100	100	100	100	100	100	100	100
n	171	284	291	361	398	395	413	254	176	235	208	195	123	104	83	50

Percentile ranks for females, separately for low and high education and age groups. In the left-most column, the number of correctly solved TOL-problems is listed.

**Table 6.** Age and education adjusted normative data of the Tower of London (TOL-F) task for men

	Low education								High education							
TOL	40–44 years	45–49 years	50–54 years	55–59 years	60–64 years	65–69 years	70–74 years	75–79 years	40–44 years	45–49 years	50–54 years	55–59 years	60–64 years	65–69 years	70–74 years	75–79 years
1	0	0	0	0	0	1	0	1	0	0	0	0	0	0	0	1
2	0	0	0	0	0	1	1	2	0	0	0	0	0	0	0	1
3	0	0	0	0	0	1	1	3	0	0	0	0	0	0	0	1
4	0	0	0	0	1	1	3	5	0	0	0	0	0	1	0	4
5	1	0	0	0	1	2	5	7	0	0	0	0	1	2	0	4
6	1	1	1	1	2	4	8	12	0	0	0	0	2	2	2	8
7	2	3	1	1	4	7	13	18	0	1	1	0	3	6	7	17
8	4	4	3	5	8	12	18	26	1	1	2	3	3	10	13	24
9	7	7	5	10	11	18	24	33	3	3	4	7	6	15	22	31
10	9	12	8	16	19	27	34	43	4	6	6	10	10	19	30	41
11	14	18	16	22	26	37	46	53	8	7	12	14	15	24	38	52
12	17	27	25	32	35	46	59	63	12	12	18	20	24	32	49	63
13	21	33	35	40	44	58	71	72	19	18	25	29	33	47	66	73
14	33	44	43	55	56	71	80	81	26	26	37	36	48	55	76	78
15	43	57	57	67	68	82	87	88	38	36	49	48	60	65	83	86
16	59	67	67	78	79	88	93	94	50	50	61	63	70	77	92	89
17	69	79	79	88	86	95	98	97	61	62	72	72	82	87	95	96
18	80	84	90	93	93	97	99	99	71	72	81	82	90	93	98	99
19	87	92	93	97	96	98	99	100	83	81	86	93	94	97	99	100
20	94	97	97	99	99	100	100	100	92	90	95	96	97	99	100	100
21	97	99	99	100	100	100	100	100	98	98	97	99	100	100	100	100
22	99	100	100	100	100	100	100	100	99	99	98	100	100	100	100	100
23	100	100	100	100	100	100	100	100	100	100	100	100	100	100	100	100
24	100	100	100	100	100	100	100	100	100	100	100	100	100	100	100	100
n	121	205	274	273	313	312	357	247	226	249	316	287	251	216	175	140

Percentile ranks for males, separately for low and high education and age groups. In the left-most column, the number of correctly solved TOL-problems is listed.

## DISCUSSION

As expected, the effects of age, education level, and sex on Planning Accuracy replicate the results of Kaller et al. (2016) who also explicitly discussed these effects. These results also concur well with the findings of D’Antuono (2017) and Boccia et al. (2017) who reported the same effects with similar effect sizes in large Italian samples. Apparently, effects of these demographic variables are quite comparable and may thus be generalizable at least to Western Europe. As performance in the TOL is associated to sociodemographic and economic factors such as education, whether our normative data can be generalized to other samples from other societies will likely depend on their comparability with respect to such factors. Moreover, especially regarding the age-related performance trajectory, the public health system will most likely play an additional role, given the impact of cardiovascular factors such as elevated blood pressure and cardiac disease on TOL performance and their increasing prevalence in older adult age (Gold et al., 2005; Jefferson, Poppas, Paul, & Cohen, 2007).

The reason behind the triple-interaction of *Age Group* by *Education Level* and by *Sex* is not easily accounted for and any attempts to do so are highly speculative. Considering the small F-value and low effect size in such a large sample, one can question the meaningfulness of this effect. More importantly, findings like this clearly demonstrate the necessity of a fine-grained standardization of the test.

The reliability estimates were adequate and we could repeatedly show that estimates were highest for *glb* and *λ*_*4*_, whereas *λ*_*3*_ or Cronbach’s *α* yielded the lowest value in all cases. This ordering of reliability estimates for the TOL-F is in line with those observed for a non-computerized four-disc TOL variant (TOL-4D) recently put forward by Tunstall et al. (2016). However, reliability estimates of the TOL-4D in adults were substantially lower reaching only a glb of .65, a λ4 of .56, a ω_tot_ (omega) of .35, and a Cronbach’s α of .27. Thus, although the current reliability estimates clearly exceed those of the TOL-4D, both findings strongly conform with the argument of Sijtsma (2009) that the λ3 measure (or Cronbach α) often constitutes a gross underestimate. Thus, Sijtsma (2009) recommended *glb* as a better alternative that was extensively discussed in the earlier reports (Kaller, Unterrainer, Kaiser, et al., 2012; Kaller et al., 2016).

Obviously, reliability estimates of the TOL-F remained stable on this level as they only minimally changed compared to the data of Kaller et al. (2016), even though estimates in that study was based on roughly half of the current sample size (Kaller et al., 2016; *n*=3770; current study: *n*=7703). Taken together, these results suggest that the TOL-F features an adequate and satisfactory reliability with estimates based on *glb* and *λ*_*4*_ attaining values close to or exceeding .7 for the overall sample as well as for all age-groups (Table 3). Thus, the TOL-F succeeded in overcoming the seemingly contradictory demands of providing items that rely on novel situations to overcome routine behavior as defined for executive functions on the one hand, and to offer a homogeneous, limited set of problems exhibiting sufficient reliability on the other hand.

As one could expect from the strong main effects of the reported ANOVA above, there are notable differences in the distribution of the percentiles in the normative data depending on age, education, and sex. To give an example: If a highly educated man aged 45 solved 50%, that is, 12 of the 24 TOL problems correctly, he only scores at the 12th percentile. Assuming values equal to or greater than the 16th percentile as the lower end of the normal performance range, his planning ability can be rated as below average. In contrast, a 45-year-old man with low education and 12 correctly solved problems reaches the 27th percentile and is thus well in the normal range.

Please note that even in most subgroups of the sex-separated normative data the number of subjects reaches a minimum of 100 to 150 and thus meets the demands for an excellent sample size as suggested by the EFPA (2013). Only for the highly educated female participants, cell sizes for the two oldest subgroups (ages 70 to 80 years) are considerably lower (83 and 50, respectively), although the recruitment of the GHS study set highest standards to cover a representative population based sample. This presumably reflects the more limited access of females to higher education compared to males 70 or 80 years ago.

### Limitations

When translating these results to other TOL studies, one has to consider some special characteristics of the TOL-F version used in the presented study. First, single items were time-limited to 1 min. Although Tim Shallice used the same time restriction in his original version, other versions have longer (e.g., Culbertson & Zillmer, 1998; Schnirman et al., 1998; 2 min for each trial) or no time limits reported (e.g., Krikorian et al., 1994), respectively. Second, participants had to solve the problems using a touch-screen, not by computer mouse. Especially among the older participants, many were unexperienced in computer use and handled a computer mouse for the first time at the beginning of the GHS. Thus, we switched to using a touchscreen version, which has proven feasible and advantageous for elderly participants. Third, an overall time limit of 20 min was introduced, mainly to avoid delays in the subjects’ schedules at the GHS. As available time is a very critical issue in clinical assessment, this overall testing limitation should guarantee the tasks’ suitability for both research and clinical application. Cancellation rates due to the 20-min limit even in the oldest subgroups remained rather low (max. 14%; Table 2) justifying this consideration.

Moreover, there was no bias with respect to education level, unduly constraining participants with lower education, which might have led to an underestimation of their performance. As we cannot avoid age-related slowing from impacting performance, we provide age group-wise normative data, hereby ensuring a presumably less biased basis for comparisons across ages.

## CONCLUSION

The TOL-F was shown to possess adequate psychometric properties that are stable across the adult life span. The 24-item version covers a broad range of graded difficulty even in healthy adults, which makes this task suitable for both research and clinical application. The reported normative data enable assessment of individual planning performance compared to a comprehensive representative age-, sex-, and education-fair sample. This in combination with use of a computerized task version should ease and standardize the use of the Tower of London task. We thank an unknown reviewer who commented that “This is an important time in the field of neuropsychology as the need to use technology to improve our assessments is vital to the sustainability of the field. You need to show that technology is an inclusive model for assessing all individuals and provide sufficient information to pass the high level of scrutiny that computerized tests will endure from clinicians.” We hope that our study could help to support this development in neuropsychological assessment.
